# Tolerability of glutamine supplementation in older adults: a double-blind placebo-controlled randomized clinical trial

**DOI:** 10.1590/1414-431X2024e13468

**Published:** 2024-05-20

**Authors:** T.C.M. de Nóbrega, M.A.R.C.P. da Silva, E.M. Rampani, R. Curi, R.B. Bazotte

**Affiliations:** 1 Programa de Pós-graduação Interdisciplinar em Ciências da Saúde, Universidade Cruzeiro do Sul, São Paulo, SP, Brasil; 2 Departamento de Farmacologia e Terapêutica, Universidade Estadual de Maringá, Maringá, PR, Brasil; 3 Programa de Pós-graduação em Ciências Farmacêuticas, Universidade Estadual de Maringá, Maringá, PR, Brasil; 4 Seção de Produção de Imunobiológicos, Centro Bioindustrial, Instituto Butantan, São Paulo, SP, Brasil

**Keywords:** Nutraceuticals, Nutrients, Cytokines, Aging, Amino acids

## Abstract

In this double-blind placebo-controlled randomized investigation, we assessed the tolerability of glutamine in older adults recruited from three daycare centers. The relevance of studying glutamine supplementation in elderly patients lies in its potential to provide a well-tolerated intervention. Glutamine, a crucial amino acid, plays a vital role in various physiological processes, including immune function and protein synthesis. Understanding its impact on older adults is essential, given the potential implications for their health and well-being. Participants received a daily dose of 12.4 g of oral effervescent glutamine (EGln group) or maltodextrin (placebo group) for 60 days. Fifteen patients from each group completed the study. The mean ages were 77.0±9.1 and 79.0±6.9 years for the EGln and placebo groups, respectively. We evaluated body mass index, aminogram, hemogram, plasma levels of glucose, prealbumin, albumin, urea, creatinine, uric acid, C-reactive protein, vitamin D, calcium, sodium, potassium, and the plasma activities of aspartate aminotransferase and alanine aminotransferase. Notably, we quantified a broad array of inflammatory markers and growth factors providing a holistic understanding of the potential effects of glutamine supplementation. The results demonstrated that oral glutamine did not induce significant changes in any evaluated parameters, and no adverse effects were reported. This finding suggested that the dosage of glutamine used in this study was well-tolerated and safe. This information contributes to the broader understanding of glutamine supplementation, emphasizing its safety and supporting its potential as a viable intervention for maintaining health in aging individuals.

## Introduction

Aging is characterized by a gradual decline in the ability to adapt to the environment, a reduction in social standing, diminished functional capacity and independence, increased susceptibility to infections, a higher prevalence of chronic diseases, and an elevated risk of morbidity and mortality (1,2). Additionally, normal aging is marked by a low-grade chronic systemic inflammation, evident through elevated blood levels of inflammatory biomarkers (3,4). This inflammatory state is intricately linked to conditions such as insulin resistance, cardiovascular diseases, type 2 diabetes, and neurodegenerative diseases (5).

Moreover, aging is intricately associated with a constellation of factors, including immunosenescence, cognitive decline, reduced social interactions, diminished physical activity, decreased grip strength, sarcopenia, osteopenia, abnormal baroreflex sensitivity, postural instability, an increased risk of falls, slowness, fatigue, poor responses to stress, anorexia, anemia, the presence of chronic diseases, heightened susceptibility to illness, hospitalization, and death (6-10). Various elements such as widowhood, lower income, limited educational attainment, a sedentary lifestyle, and alcoholism contribute to a deteriorating quality of life during normal aging (senescence), resulting in abnormal aging (senility), and frailty with increased healthcare costs (11-13).

As the global population of older individuals continues to grow, it is crucial to develop strategies that can delay, alleviate, or even reverse the processes associated with senescence, senility, and frailty.

Despite the potential beneficial effects of glutamine, including activating the immune response, stimulating protein anabolism, protecting the intestinal barrier function, promoting an anti-inflammatory response (14-21), and providing protection against oxidative stress (21), there is a limited number of studies involving glutamine supplementation in older adults (18,19,22). Additionally, there is a lack of investigations into the tolerability of this amino acid among the older adult population. Our hypothesis was that glutamine is safe for older adults, as previously established for younger patients (15,23-26). To test this hypothesis, we conducted a double-blind placebo-controlled randomized study comparing older adults receiving oral effervescent glutamine (EGln) or maltodextrin (placebo) for 60 days.

## Material and Methods

### Ethical approval

The protocol received approval from the State University of Maringá Standing Committee on Human Subject Research Ethics (COPEP 1.808.919).

### Participants

We recruited forty-four individuals from three daycare centers registered with the Council for the Rights of Older Adults in Maringá, Brazil.

The eligibility criteria required participants to be: 1) aged >60 years and 2) willing to participate in the study. The geriatric doctor on our team (T.C.M.N.) made the final decision to include each patient, following an evaluation of their potential for treatment engagement, medical history, and lab test results.

Exclusion criteria were 1) recent infectious disease; 2) HIV/AIDS, 3) inflammatory disease; 4) autoimmune disease; 5) immunosuppressant, corticosteroid, and non-steroidal anti-inflammatory drug use; 6) severe hepatic disease; 7) cancer; 8) terminal illness; 9) abnormal laboratory parameters (hemoglobin below 12 mg/dL, leukocytes exceeding 15,000/mL or below 1,500/mL, platelets below 100,000/mL, C-reactive protein exceeding 5 mg/dL, creatinine clearance below 30 mg/mL per min).

Thirty-four individuals or their legal representatives completed, signed, and dated the informed consent form. Before initiating treatment, all patients underwent a clinical evaluation.

### Patient randomization

To minimize potential biases in result interpretation and provide reliable evidence about the effectiveness of the medical intervention, a double-blind placebo-controlled randomized clinical trial was implemented. This ensured that both participants and researchers involved in the study were unaware of who was receiving EGln and who was receiving the placebo.

The enrolled participants were randomly assigned to two groups, each comprising 17 volunteers. This randomization process was employed to ensure that any observed differences between the groups were not influenced by pre-existing factors but rather resulted from the studied intervention.

The control group received a placebo substance (maltodextrin), and this group was then compared to the group receiving EGln. Both substances were packaged in sachets (12.4 g). Prior to ingestion, EGln and maltodextrin were dissolved in 200 mL of water, resulting in a concentration of 62 mg/mL. EGln and maltodextrin were consumed daily for 60 days. On Fridays, each patient received two sachets for use over the weekend. Maltodextrin was selected as the control substance due to its low cost, easy preparation, high solubility in water, and its prior utilization in our previous clinical study (15).

Body mass index (BMI) was evaluated before and after 60 days of EGln or maltodextrine supplementation.

### Clinical evaluation and characterization of the participants

A questionnaire was administered to collect information on age, gender, race, medical history, educational level, marital status, therapeutic profile, and lifestyle.

To assess physical activity levels in adults the International Physical Activity Questionnaire (IPAQ) was utilized (27). The IPAQ is structured to provide a comprehensive overview of a person's physical activity patterns across various domains of daily life, including work, transportation, household chores, and leisure-time activities. The key components measured by the IPAQ include intensity, duration, and frequency of physical activity.

The Charlson Comorbidity Index is a scoring system that provides a standardized and quantitative measure of comorbidities, allowing healthcare professionals to assess the impact of these conditions on patient prognosis (28).

To evaluate how well older individuals can perform basic everyday activities on their own (bathing, dressing, moving in and out of bed or a chair, control of bowel and bladder function, using the toilet, and ability to eat independently) the Katz Index of Independence in Activities of Daily Living was used (29). This index helps us understand both the physical and mental aspects of their well-being.

The Lawton & Brody Scale (30) includes the self-maintaining activities of daily living (SADL) and instrumental activities of daily living (IADL) subscales. SADL refer to basic self-care and physical well-being (bathing, dressing, grooming, and control over bowel and bladder). IADL involves more complex tasks that are crucial for independent living in the community (cooking, shopping, managing finances, using transportation, and performing household chores, etc.). Therefore, IADLs reflect a person's ability to function independently in a broader social context. These activities are important for maintaining a person's quality of life and ability to live in a community setting.

The International Clinical Practice Guidelines for Identification and Management of Physical Frailty provides a comprehensive approach to identify and manage physical frailty, emphasizing personalized and multidimensional strategies encompassing physical, nutritional, psychological, and social aspects (31). These strategies include questionnaires, physical performance tests, and other measures. Geriatricians play a crucial role in assessing an individual's overall health and medical history and conducting physical examinations to identify signs of frailty (weakness, slow walking speed, unintentional weight loss, low physical activity, and fatigue).

### Preparation of EGln

Each sachet contained 12.5 g of glutamine (Ajinomoto North America, USA), combined with 3.90 g of sodium bicarbonate as the effervescent base (Arbros Industria Pharma e Alimentícia Ltda., Brazil), as well as 1.30 g citric acid and 2.60 g of tartaric acid (SM Pharmaceutical Enterprises, Brazil), which release carbon dioxide when dissolved in water to initiate effervescence (32).

### Blood collection

Blood was collected after an overnight fast. Immediately after blood collection, a portion of fresh blood was used to measure glycated hemoglobin A1c and a complete blood count. Another portion of fresh blood was transferred to a tube containing sodium fluoride/EDTA, which was immediately centrifuged (2000 *g* for 10 min at room temperature), and the plasma was separated for measurements of amino acid, biochemical parameters, and cytokines.

### Plasma aminogram

Plasma levels of argininosuccinic acid, aspartic acid, glutamic acid, alanine, arginine, citrulline, glycine, histidine, leucine, isoleucine, methionine, ornithine, phenylalanine, proline, asparagine, serine, tyrosine, threonine, tryptophan, and valine were measured by high-performance liquid chromatography (Hermes Pardini Laboratory, Brazil).

### Hematological parameters

A BC Plus 3000 Hematology Analyzer (Mindray, USA) was used to measure hemoglobin, hematocrit, mean corpuscular hemoglobin concentration (MCHC), leukocytes, platelets, segmented cells, lymphocytes, monocytes, eosinophils, and basophils.

### Biochemical parameters

Blood levels of glycated hemoglobin A1c, plasma concentrations of glucose, prealbumin, albumin, urea, creatinine, uric acid, C-reactive protein, vitamin D, calcium, sodium, potassium, and plasma activities of aspartate aminotransferase (AST) and alanine aminotransferase (ALT) were determined by lab tests using conventional kits according to the manufacturer's recommendations.

### Plasma cytokines

We quantified B lymphocyte chemoattractant (BLC) CXCL13 (BLC/CXCL13), eosinophil chemotactic protein (Eotaxin), Eotaxin-2, growth-regulated oncogene protein-α (GRO-α), hepatocyte growth factor (HGF), interleukin (IL)-1β, IL-2, IL-3, IL-7, IL-9, IL-15, IL-17α, IL-18, interferon-inducible T-cell α chemoattractant (I-TAC), monocyte chemotactic protein-1 (MCP-1), macrophage inflammatory protein-1α (MIP-1α), MIP-3α, IFN-gamma-inducible protein 10 (IP-10), macrophage migration inhibitory factor (MIF), matrix metalloproteinase-1 (MMP-1), stem cell factor (SCF), stromal cell-derived factor 1α, (SDF-1α), soluble CD40 ligand (SCD-40L), tumor necrosis factor α (TNF-α), and vascular endothelial growth factor α (VEGF-α). We used the human cytokine Magnetic Plex Panel by Invitrogen^TM^ (USA) in conjunction with the Luminex-Magpix^®^ (USA) immunoassay platform.

### Statistical analysis

Analysis of variance (ANOVA) and the Tukey test for multiple comparisons were used to assess the differences in results within each group (EGln group or placebo group) on days 0, 30, and 60. Additionally, an unpaired Student's *t*-test was used to compare results between the EGln and placebo groups on the aforementioned days. The statistical analyses was performed using Statistica^TM^ 8.0 software (StatSoft, Germany; https://www.statistica.com). Results are reported as means±SD. A significance level of P<0.05 was considered statistically significant.

## Results

The mean±SD age for the EGln group was 77.0±9.1 years, ranging from 64 to 91 years, while for the placebo group, it was 79.0±6.9 years, ranging from 61 to 88 years. Each group had eleven women and four men. In the EGln group, there was one married, three divorced, and eleven widowed individuals, while in the placebo group, there was one married, one divorced, two single, and eleven widowed individuals. The level of education in both groups ranged from 0 to 8 years, with a mean±SD of 2.7±2.7 years for the EGln group and 1.9±2.3 years for the placebo group.

As conveyed by the geriatric specialist on our team (T.C.M.N.), all participants were of Caucasian ethnicity. In the EGln group, there was 1 smoker, 10 hypertensive individuals, and 4 diabetics while the placebo group had 13 hypertensive subjects and 4 diabetics. The average number of medications used by patients in the EGln group was 4.3 (ranging from one to eleven medications), whereas in the placebo group it was 5.7 (ranging from zero to seventeen medications).

Based on the IPAQ, all volunteers from both the EGln and placebo groups exhibited low levels of physical activity.

The Charlson Comorbidity Index estimated a lifespan of ten years for both groups, ranging from 2 to 77% for the ELGn group and 0 to 77% for the placebo group.

According to the Katz Index of Independence in Activities of Daily Living, nine individuals in the EGln group were classified as independent, four as partially dependent, and two as dependent. In the placebo group, nine were independent, six were partially dependent, and none were completely dependent.

The Lawton & Brody Scale revealed that two individuals in the EGln group were independent, seven partially dependent, and six dependent. In the placebo group, three were independent, six were partially dependent, and six were dependent.

Using the International Clinical Practice Guidelines for Identification and Management of Physical Frailty, we identified nine pre-frailty and six frailty cases in the EGln group and ten pre-frailty and five frailty cases in the placebo group.

During the treatment period, one patient from the placebo group passed away and three dropped out (one from placebo group and two from EGln group), while fifteen individuals each from the EGln group (n=15) and the placebo group (n=15) successfully completed the clinical trial. Furthermore, no participant reported any discomfort (such as nausea, dyspepsia, or other undesirable effects) during glutamine supplementation.

The BMI values before and after supplementation with EGln were 29.1±5.3 and 29.3±5.5, respectively. Similarly, BMI values before and after supplementation with maltodextrin (placebo) were 30.3±5.3 and 30.1±5.1, respectively. These results indicated that EGln and maltodextrin did not induce any significant change in BMI.

With the exception of threonine, the plasma levels of various amino acids (argininosuccinic acid, aspartic acid, glutamic acid, alanine, arginine, citrulline, glycine, histidine, leucine, isoleucine, methionine, ornithine, phenylalanine, proline, asparagine, serine, tyrosine, tryptophan, and valine) did not show statistically significant differences between the EGln and placebo groups on day 0 (before supplementation with EGln or maltodextrin) and on day 60 (after supplementation with EGln or maltodextrin) (Table 1).

**Table 1:** Plasma levels of amino acids in older adults before (day 0) and after (day 60) treatment with the effervescent glutamine formulation (EGln) or placebo (PL).

Amino acids	Groups	Day 0 (μmol/L)	P-value	Day 60 (μmol/L)	P-value
Argininosuccinic acid	EGln	1.50±0.66	0.110534	1.8±1.11	0.150353
	PL	1.15±0.38		1.3±0.49	
Aspartic acid	EGln	25.0±6.77*	0.031214	25.8±4.06	0.056728
	PL	19.1±6.37		22.0±5.51	
Glutamic acid	EGln	102.3±25.0	0.471956	110.7±27.3	0.094799
	PL	94.4±29.9		94.6±19.2	
Alanine	EGln	148.2±65.3	0.105270	156.9±50.4	0.218604
	PL	110.5±47.5		133.8±42.5	
Arginine	EGln	49.7±21.4	0.099653	50.4±13.8	0.091170
	PL	37.5±14.2		40.7±14.3	
Citrulline	EGln	23.8±10.9	0.121496	23.1±8.6	0.343017
	PL	17.7±8.3		18.6±14.4	
Glycine	EGln	148.2±55.6	0.088712	156.0±57.7	0.255485
	PL	114.0±41.7		128.8±61.3	
Histidine	EGln	70.0±31.0	0.923812	71.3±24.7	0.533239
	PL	71.2±32.3		65.2±24.5	
Leucine + Isoleucine	EGln	74.5±26.3	0.069755	80.7±20.5	0.105396
	PL	57.2±19.7		66.7±21.9	
Methionine	EGln	14.9±6.9	0.241177	17.0±8.4	0.208397
	PL	12.0±5.3		13.6±4.4	
Ornitine	EGln	53.8±20.5*	0.045425	54.9±14.5	0.095082
	PL	39.0±14.8		44.6±15.7	
Phenylalanine	EGln	41.1±13.7	0.063482	43.0±11.1	0.253482
	PL	31.2±12.2		37.1±14.4	
Proline + Asparagine	EGln	366.2±110.0	0.590355	411.9±136.1	0.417629
	PL	339.6±137.1		370.5±119.3	
Serine	EGln	33.44±15.3*	0.022152	37.1±17.7	0.188374
	PL	22.9±2.7		28.6±14.11	
Tyrosine	EGln	40.0±16.3	0.047364	44.4±16.0	0.080949
	PL	28.3±11.9		33.7±13.9	
Threonine	EGln	22.9±9.5	0.194832	25.6±7.6*	0.018958
	PL	18.6±6.7		18.0±7.8	
Tryptophan	EGln	27.3±8.4	0.678935	30.6±8.1	0.073244
	PL	25.8±9.8		25.3±6.2	
Valine	EGln	97.8±34.9	0.063287	98.9±19.7	0.051501
	PL	74.1±26.6		81.6±23.2	

Data are reported as means±SD of three analyses (n=12-13/group). *P<0.05, unpaired Student's *t*-test was used to compare the results between the EGln and placebo groups on days 0 and 60.

Except for hematocrit, the blood parameters hemoglobin, MCHC, leukocytes, platelets, segmented cells, lymphocytes, monocytes, eosinophils, and basophils all exhibited values within the normal range before and after 60 days of supplementation with either EGln or maltodextrin (Table 2).

**Table 2:** Hematological parameters in older adults before (day 0) and after 30 (day 30) and 60 days (day 60) of daily supplementation with 12.4 g of effervescent glutamine (EGln group) or 12.4 g of maltodextrin (PL group).

Hematological parameters	Groups (n=15)	Day 0	P-value	Day 30	P-value	Day 60	P-value	F-value
Hemoglobin (g/dL)	EGln	12.9±0.6	>0.999	13.0±0.46	0.9879	13.2±0.59	0.9879	0.2509
	PL	12.9±1.0		13.1±1.23		13.1±1.34		
Hematocrit (%)	EGln	39.3±1.7	0.9884	39.3±1.14	0.9996	42.9±1.36*	0.0047	4.049
	PL	39.0±3.2		39.4±3.60		39.5±4.40		
MRCV (fL)	EGln	90.6±3.6	0.9664	90.6±4.00	0.9853	91.1±3.68	0.9664	0.1345
	PL	91.4±6.3		91.2±6.28		91.9±6.52		
Leukocytes (cells/mL)	EGln	6380±1167	0.4051	7140±1339	0.9931	6426.7±1152	0.9842	0.6607
	PL	7329±2785		6979±1795		6764.3±2142		
Platelets (cells/mL)	EGln	211000±52368	0.4224	212866±46774	0.9778	214733±4007	0.8274	1.808
	PL	238000±59897		245000±57695		252928±5871		
Segmented (cells/mL)	EGln	3476±1022	0.3975	3754.6±1013.2	0.8940	3208.1±891.7	0.8296	1.119
	PL	4178±1952		4065.6±1329.6		3583.0±1525		
Lymphocytes (cells/mL)	EGln	2620±468	0.5351	2676.3±653.2	0.2059	2622.5±560.8	0.9998	1.424
	PL	2313±728		2222.6±892.2		2241.1±737.4		
Monocytes (cells/mL)	EGln	320±72	0.5462	426.3±183.4	0.5462	379.7±112.0	0.6770	0.9998
	PL	381±146		413.2±77.5		464.9±195.9		
Eosinophils (cells/mL)	EGln	2305±135.1	0.9792	282.9±178.0	0.8751	216.3±143.9	0.9837	0.2236
	PL	255±275		236±201		238.7±167.3		
Basophils (cells/mL)	EGln	0.0±0.0		0.0±0.0		0.0±0.0		
	PL	0.0±0.0		0.0±0.0		0.0±0.0		

Data are reported as means±SD (n=15/group). MRCV: Mean red cell volume. *P<0.05, ANOVA with Tukey test was used for multiple comparisons to assess the differences in results within each group on days 0, 30, and 60.

In general, the plasma levels of BLC/CXCL13, eotaxin, eotaxin-2, GRO-α, HGF, IL-1β, IL-2, IL-3, IL-7, IL-9, IL-15, IL-17α, IL-18, I-TAC, MCP-1, MIP-1α, MIP-1β, MIP-3α, IP-10, MIF, MMP-1, SCF, SDF-1α, SCD-40L, TNF-α, and VEGF-α remained unchanged after 60 days of oral supplementation with EGln. However, there were two exceptions: ITAC and MIF levels decreased in the placebo group between day 0 and day 60 (Table 3).

**Table 3:** Plasma cytokine levels (pg/mL) in older adults before (day 0) and after (days 30 and 60) supplementation with effervescent glutamine (EGln) or placebo (PL) at a daily dosage of 12.4 g.

Cytokines	Groups (n=15)	Day 0	P-value	Day 30	P-value	Day 60	P-value	F-value
BLC/CXCL13	EGln	162.2±32.6	0.0519	175.5±24.3	0.2999	134.1±19.3	<0.0001	<0.0001
	PL	143.5±15.4		138.8±9.8		146.5±18.0		
Eotaxin	EGln	163.7±20.1	0.1266	183.4±19.2	0.9594	179.3±24.6	0.9994	1.760
	PL	178.8±14.3		180.1±16.4		179.3±24.6		
Eotaxin 2	EGln	315.9±72.5	0.9996	331.6±87.5	0.0277	321.5±24.4	0.1346	2.439
	PL	313.9±47.8		276.4±43.9		279.7±40.4		
GRO-α	EGln	2.94±0.9	0.8394	5.68±1.5	0.0076	2.72±0.98	<0.0001	22.35
	PL	2.56±0.6		4.10±0.99		6.61±2.5		
HGF	EGln	223.9±23.5	0.4511	257.7±35.2	0.0007	188.9±22.3	0.2516	10.14
	PL	238.3±35.7		216.6±25.3		207.1±30.5		
IL-1β	EGln	5.36±0.7	0.9862	5.80±0.9	0.0001	5.8±0.46	0.0710	11.10
	PL	5.24±0.6		7.52±2.2		4.9±0.46		
IL-2	EGln	22.0±4.3	<0.0001	24.2±6.1	<0.0001	18.5±3.0	0.0415	16.52
	PL	16.3±1.3		16.4±1.2		15.4±0.94		
IL-3	EGln	12.7±2.1	<0.0001	13.3±1.1	0.6700	10.6±1.1	0.2133	15.07
	PL	17.1±3.5		14.1±2.0		12.7±2.1		
IL-7	EGln	4.1±1.1	0.0388	3.4±0.8	0.0048	2.2±0.4	0.0004	12.45
	PL	3.4±0.56		2.5±0.33		3.3±1.0		
IL-9	EGln	39.0±10.0	<0.0001	37.1±10.1	<0.0001	34.7±8.0	0.0257	15.54
	PL	19.8±4.6		22.8±4.2		27.0±8.0		
IL-15	EGln	10.1±3.9	0.0032	10.5±4.1	<0.0001	7.9±2.3	0.0085	11.14
	PL	6.9±1.1		5.9±0.77		5.0±0.98		
IL-17α	EGln	794±149.7	<0.0001	675±115	0.0005	630±117.4	0.1031	18.35
	PL	459.3±65.4		516±78.5		544.2±113.2		
IL-18	EGln	29.2±8.2	<0.0001	23.7±7.56	<0.0001	15.7±5.5	0.9176	23.83
	PL	13.8±3.1		10.1±1.60		16.87±4.2		
IP-10	EGln	50.4±8.6	0.5461	86.3±31.2	<0.0001	40.58±6.4	0.0003	16.82
	PL	56.77±6.4		60.5±8.7		62.2±7.2		
I-TAC	EGln	61.6± 9.0	<0.0001	58.0±6.20	<0.0001	33.0±11.7	<0.0001	58.17
	PL	87.0±7.5		75.7±10.9		53.3±10.4*		
MCP-1	EGln	120±19.9	<0.0001	117.3±16.7	<0.0001	103.8±17.1	<0.0001	38.74
	PL	185±33.8		197±31.0		179.7±29.0		
MIF	EGln	41.9±3.6	<0.0001	41.9±3.20	0.2518	35.5±2.7	0.9104	41.39
	PL	50.3±4.6		39.9±1.90		36.2±2.6*		
MIP-1α	EGln	55.8±16.4	0.0005	56.6±14.3	0.1414	15.91±3.1	0.1864	50.56
	PL	72.7±13.6		48.1±10.6		23.88±6.8		
MIP-3α	EGln	29.9±5.3	0.0070	27.28±4.7	<0.0001	23.21±3.7	0.4548	9.277
	PL	25.2±3.1		20.55±2.5		25.22±4.6		
MMP-1	EGln	607±158.2	0.9994	459.8±107.1	0.9653	440.6±02.7	0.0016	9.396
	PL	611±142.4		443.4±100.8		580.5±29.9		
SCD40L	EGln	78.74±21.0	<0.0001	72.4±20.1	0.0005	48.1±15.7	<0.0001	25.59
	PL	115.1±17.1		98.3±13.4		96.5±19.7		
SCF	EGln	24.96±4.7	<0.0001	20.4±3.8	0.1533	21.7±5.1	0.9959	8.272
	PL	16.9±2.5		17.5±2.5		22.0±4.9		
SDF1	EGln	91.8±37.3	0.2625	88.8±23.6	0.0009	84.3±53.0	0.9792	7.493
	PL	73.9±16.1		48.5±6.4		44.74±6.8		
TNF-α	EGln	12.8±4.5	0.0062	16.6±4.9	<0.0001	6.2±1.0	>0.9999	25.47
	PL	9.2±2.2		11.29±1.8		6.2±2.1		
VEGF-α	EGln	57.9±24.7	0.0092	39.3±7.4	0.9361	28.6±5.6	0.2197	9.333
	PL	44.0±11.7		36.9±7.8		36.7±6.5		

BLC/CXCL13: B lymphocyte chemoattractant CXCL13; Eotaxin: eosinophil chemotactic protein; GRO-α: growth-regulated oncogene protein-α; HGF: hepatocyte growth factor; IL: interleukin; I-TAC: interferon-inducible T-cell α chemoattractant; MCP-1: monocyte chemotactic protein-1; MIP: macrophage inflammatory protein; IP-10: IFN-γ-inducible protein; MIF: macrophage migration inhibitory factor; MMP: matrix metalloproteinase; SCF: stem cell factor; SDF-1α: stromal cell-derived factor 1α soluble: SCD-40L: CD40 ligand; TNF-α: tumor necrosis factor α; VEGF-α: vascular endothelial growth factor α. Data are reported as means±SD (n=15/group). *P<0.05 between day 60 and day 0, ANOVA with the Tukey test for multiple comparisons was used to evaluate variations in results within each group across days 0, 30, and 60.

Fasting glycemia and glycated hemoglobin A1c values before and after 60 days of supplementation with EGln or maltodextrin indicated prediabetes, but no significant differences were observed between the EGln and placebo groups. Additionally, the plasma levels of prealbumin, albumin, urea, creatinine, uric acid, PCR, vitamin D, calcium, sodium, potassium levels, and plasma activities of AST and ALT all exhibited normal range and similar values on days 0, 30, and 60 in both the EGln and placebo groups (Table 4).

**Table 4:** Biochemical and toxicological parameters in older adults before (day 0), after 30 (day 30), and after 60 days (day 60) of daily supplementation with 12.4 g of effervescent glutamine (EGln group) or 12.4 g of maltodextrin (PL group).

Parameters	Groups (n=15)	Day 0	P-value	Day 30	P-value	Day 60	P-value	F-value
Glucose (mg/dL)	EGln	103.5±25.1	0.8054	102.9±19.6	>0.9999	107.0±19.9	0.8949	0.4651
	PL	110.4±26.6		103.0±22.5		112.4±25.4		
HbA1c (%)	EGln	6.03±1.34	>0.9999	6.17±1.06	>0.9999	6.06±0.93	0.9916	0.08823
	PL	6.03±0.77		6.19±0.74		6.15±0.73		
Pre-albumin (mg/dL)	Egln	0.23±0.03	0.7035	0.22±0.04	0.0504	0.23±0.04	0.1559	2.226
	PL	0.25±0.06		0.27±0.07		0.27±0.08		
Albumin (g/dL)	EGln	3.99±0.29	0.6150	3.79±0.18	0.8457	4.21±0.35	0.0699	10.10
	PL	4.11±0.31		3.87±0.19		4.46±0.40		
Urea (mg/dL)	EGln	43.5±11.2	0.9855	49.8±17.9	0.5976	50.5±11.6	0.1208	1.233
	PL	45.1±14.6		44.0±11.1		39.9±16.4		
Creatinine (mg/dL)	EGln	0.92±0.20	0.9855	0.99±0.25	0.8503	0.98±0.20	0.9668	0.8524
	PL	0.89±0.25		1.06±0.34		1.02±0.31		
Uric acid (mg/dL)	EGln	4.85±1.28	0.9919	4.99±1.3	>0.9999	4.74±1.21	0.9960	0.07522
	PL	4.99±1.41		5.00±1.95		4.85±1.73		
Vitamin D (ng/mL)	EGln	21.4±10.7	0.4463	20.1±7.27	0.9035	27.1±9.30	0.9788	3.450
	PL	17.2±6.25		18.2±6.69		26.0±9.71		
Calcium (mmol/mL)	EGln	1.19±0.09	>0.9999	1.17±0.07	0.2367	1.18±0.07	>0.9999	0.7158
	PL	1.19±0.07		1.22±0.08		1.18±0.09		
Sodium (mEq/L)	EGln	140.9±1.1	0.4286	139.6±1.9	0.8682	137.3±1.67	0.9267	9.244
	PL	139.9±1.92		139.1±2.38		136.9±2.56		
Potassium (mEq/L)	EGln	4.72±0.41	0.9619	4.53±0.27	0.9415	4.59±0.24	0.5741	1.082
	PL	4.66±0.31		4.60±0.52		4.43±0.43		
AST (UI/L)	EGln	21.6±9.46	0.3630	24.9±8.9	0.2041	18.8±6.06	0.9840	2.017
	PL	17.9±2.46		20.4±6.19		19.6±5.21		
ALT (UI/L)	EGln	21.9±18.4	0.2472	25.7±10.7	0.9136	25.8±10.1	0.9683	2.047
	PL	15.2±5.81		23.4±7.18		24.2±6.90		
CRP (mg/L)	EGln	3.25±3.57	0.9998	4.28±3.08	0.9984	2.62±2.91	0.9652	0.5156
	PL	3.34±3.93		4.1±3.01		3.14±3.58		

Data are reported as means±SD (n=15/group). HbA1c: glycated hemoglobin A1c; AST: aspartate aminotransferase; ALT: alanine aminotransferase; CRP: C reactive protein. P>0.05, ANOVA with the Tukey test for multiple comparisons was used to evaluate variations in results within each group across days 0, 30, and 60.

## Discussion

There are few studies available on the plasma aminogram in older adults (33-36). In this study, we observed no significant changes in aminoacidemia after 60 days of EGln supplementation. Additionally, glutamic acid, the primary metabolite of glutamine, remained unchanged after 60 days of EGln supplementation.

Hematological analysis plays a crucial role in the toxicological evaluation of a substance, offering valuable insights into its potential adverse effects on blood and its components (37). In the context of this study, EGln treatment did not adversely impact hematological parameters. The exclusion of volunteers with hemoglobin levels below 12 mg/dL, leukocytes counts above 15,000/mL or below 1,500/mL, and platelet counts below 100,000/mL contributed to the maintenance of normal range values for all hematological parameters following EGln supplementation.

Despite the well-known anti-inflammatory properties of glutamine (20,21,38), we generally did not observe significant changes in the plasma levels of inflammatory biomarkers after 60 days of glutamine supplementation. The only exceptions were the reductions in plasma levels of ITAC and MIF in the placebo group from day 0 to day 60, but these changes were not clinically significant. The exclusion of volunteers with C-reactive protein levels above 5 mg/dL and those with inflammatory diseases reduced the likelihood of detecting an anti-inflammatory effect of glutamine.

Consistent with results reported for young individuals (17-19,38-40), we hypothesized that glutamine supplementation would be well-tolerated in older adult subjects. This hypothesis was confirmed, as we observed no changes in BMI, plasma levels of creatinine (indicative of renal toxicity), AST, ALT (indicative of liver toxicity), and urea (reflective of protein catabolism).

To the best of our knowledge, there exists only one comparable clinical controlled, double blind, crossover study investigating the tolerability of glutamine in older adults (23). However, that study included not only older adults but also individuals of middle age. Similar to our approach, they utilized L-glutamine dissolved in water immediately before ingestion. Nonetheless, they did not use maltodextrin, but calcium caseinate milk protein powder treated with food-grade glyceryl mono-oleate dissolved in water immediately before ingestion as the placebo group. The study conducted by Galera et al. (23) featured a cross-over study with supplementation periods limited to 14 days, with a five-day washout period between treatments. The primary difference, however, lies in the daily dose of glutamine: 0.15 mg/kg × day in our study compared to 0.50 mg/kg × day in the study by Galera et al. (23). The approximately three times higher dosage in the latter study may explain why our study indicated the safety of glutamine, whereas in their study increases in serum urea nitrogen and creatinine and decreases in estimated glomerular filtration rate were observed.

To comprehensively understand the implications for clinical practice and public health measures, further studies on the tolerability of glutamine in older adults are imperative for several compelling reasons. First and foremost, the current body of research is notably limited, with only one comparable clinical, controlled, double-blind study conducted on glutamine tolerability in this population (23). Additional studies are needed to validate and build upon the insights gleaned from the existing sparse research. Secondly, the diversity of older adults, characterized by different health conditions, medication profiles, and nutritional requirements, underscores the importance of exploring glutamine's impact on different subgroups within this population. Variables such as age, gender, health status, and nutritional needs should be considered in order to gain a more nuanced understanding of the potential effects.

A critical aspect that merits exploration is the determination of optimal and safe dosages as well as duration of glutamine supplementation for older adults. This information is vital to make recommendations and ensure that older individuals can maximize the benefits with minimal risks. Moreover, diversifying the scope of research to include various control substances and placebos can enhance our comprehension of glutamine's effects. Additionally, investigating the long-term effects of glutamine supplementation is essential to assess its safety and tolerability among older adults over time. Expanding the examination to encompass a broader array of biomarkers and health outcomes will provide a more comprehensive picture of the multifaceted effects of glutamine supplementation. This includes exploring potential benefits related to muscle health and immune function, contributing to a more holistic understanding of the supplement's impact.

Considering that older adults often undergo concurrent medication regimens, it is imperative to investigate potential interactions between glutamine ingestion and commonly prescribed medications to establish safety protocols and ensuring the well-being of older individuals. Furthermore, broadening the pool of participants to include a more diverse and representative sample of older adults will enhance the generalizability of findings to a broader population. In essence, these additional studies on glutamine tolerability in older adults are indispensable for addressing existing knowledge gaps, refining recommendations, and guaranteeing the safety and efficacy of glutamine supplementation.

A noteworthy limitation of this study was the small number of volunteers who met the inclusion criteria for participation in this clinical investigation. Despite this limitation, the findings revealed that oral supplementation with EGln was safe for older adults.
